# A rare case of small bowel volvulus after jenjunoileal bariatric bypass requiring emergency surgery: a case report

**DOI:** 10.1186/1752-1947-6-78

**Published:** 2012-03-07

**Authors:** Pranav H Patel, Alistair AP Slesser, Aoff Khalil, Oliver Bassett, KT Natarajan, Jeremy I Livingstone

**Affiliations:** 1 Department of General Surgery. Watford General Hospital, Vicarage Road, Watford, UK

## Abstract

**Introduction:**

Bariatric surgery is on the increase throughout the world. Jejunoileal bypass bariatric procedures have fallen out of favor in western surgical centers due to the high rate of associated complications. They are, however, performed routinely in other centers and as a consequence of health tourism, management of complications related to these procedures may still be encountered.

**Case presentation:**

We describe a rare case of small bowel obstruction in a 45-year-old British Caucasian woman, secondary to a volvulus of the jejunoileal anastomosis following bariatric bypass surgery. The pre-operative diagnosis was confirmed by radiology. We describe a successful surgical technique for this rare complication.

**Conclusions:**

Bariatric surgery may be complicated by bowel obstruction. Early imaging is vital for diagnosis and effective management. The use of our surgical technique provides a simple and effective approach for the successful management of this bariatric complication.

## Introduction

Bariatric surgery has been used as a treatment for obesity since the early 1960s [1]. Initial operations were malabsorptive bypass procedures, the most common being jejunoileal bypass surgery. This surgical intervention has fallen out of favor due to overriding hepatic and metabolic complications, with common procedures now being gastric banding or gastric bypass surgery [2]. Despite this, jejunoileal bypass surgery remains a popular and routinely performed surgical procedure outside of North American and European surgical centers. We describe a case of small bowel obstruction following jejunoileal bypass surgery as well as a simple and effective surgical technique.

## Case presentation

**Figure 1:**
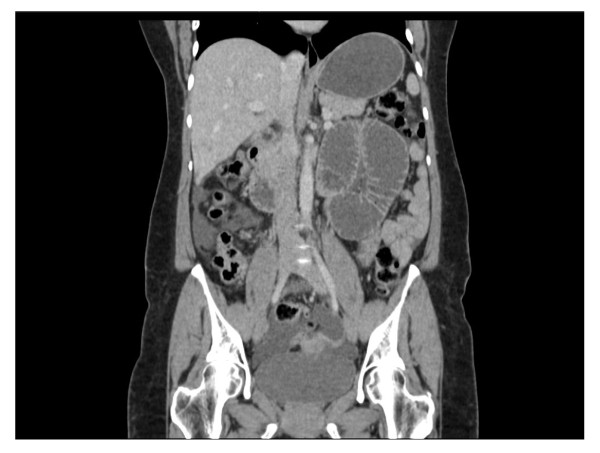
**Computed tomography of the abdomen and pelvis, coronal section**. A computed tomography scan taken on day 5, showing small bowel obstruction with proximal bowel dilatation and a transition point at the proximal jejunum.

**Figure 2:**
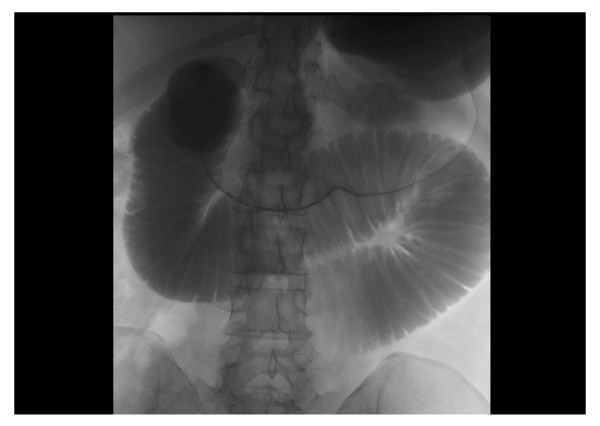
**Contrast swallow study, plain radiograph of abdomen 30 minutes after contrast ingestion**. Gastrografin contrast study demonstrating proximal jejunal dilatation with an abrupt transition point at the jejunoileal anastomosis. No further contrast passage distally, with associated collapsed small bowel.

**Figure 3:**
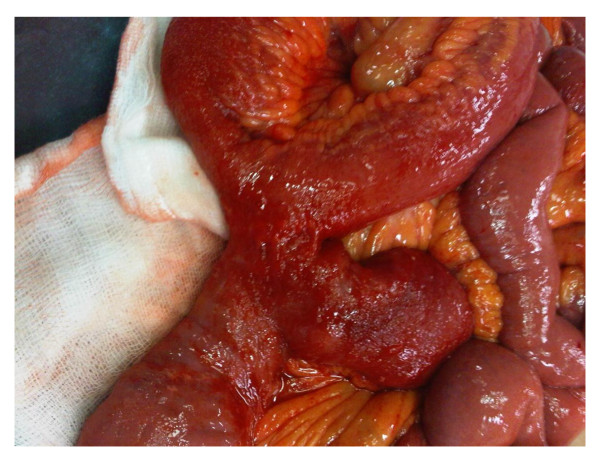
**Surgical finding: side-to-side jejunoileal anastomosis**. Intra-operative findings demonstrate a narrow jejunoileal anastomosis associated with volvulus of the bowel at the apex of anastomosis. There is dilated bowel proximally with collapsed bowel distally.

**Figure 4:**
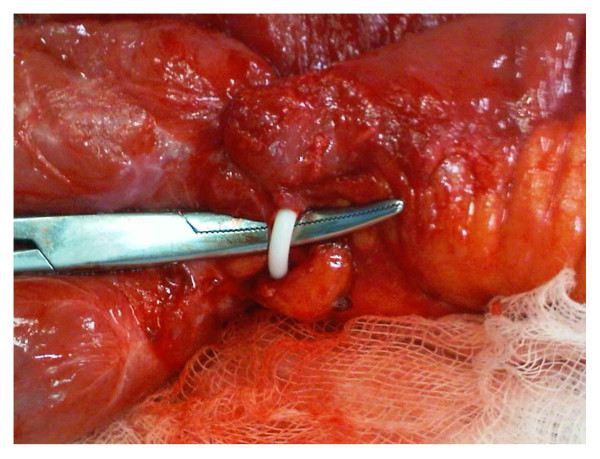
**Surgical finding: jejunal band found in small bowel mesentery**. Intra-operative finding demonstrates migration of the jejunal band into the small bowel mesentery with no associated perforation or traction injury to the bowel.

A 45-year-old British Caucasian woman with a two-day history of intermittent abdominal pain which radiated to her back presented to our surgical unit. She was opening her bowels and passing flatus infrequently. On examination, her abdomen was soft and non-distended with upper abdominal tenderness and voluntary guarding. She was hemodynamically stable and afebrile. Routine investigations revealed a hemoglobin of 13.5 g/dL, white cell count of 6900 10^9^/L, a bilirubin of 3 μmol/L and normal liver function tests. She had a background of previous open jejunoileal bypass surgery with an ileal band in 2005 with an abdominoplasty in South America. Other co-morbidities included Mobitz type one heart block with a permanent pacemaker. She also had depression. Initially, acute cholecystitis was suspected. An ultrasound scan was unremarkable. Due to the persistence of her symptoms a computed tomography (CT) of the abdomen and pelvis was performed and demonstrated small bowel obstruction with a transition point at the proximal jejunum (Figure 1). She was treated conservatively as a suspected paralytic ileus. Because her obstructive symptoms increased in severity, a gastrografin swallow study was performed that demonstrated proximal jejunal dilatation with an abrupt transition point at the jejunoileal anastomosis (Figure 2). The findings were suggestive of a small bowel volvulus at the site of anastomosis. Due to a continued deterioration in her symptoms it was decided to perform an exploratory laparotomy. Intra-operatively, a proximal small bowel volvulus at the site of the side to side jejunoileal anastomosis was found. The side-to-side anastomosis was narrow, at only 3 cm in diameter (Figure 3). The jejunal band had migrated into the small bowel mesentery with no damage to the bowel (Figure 4). The small bowel volvulus was corrected with ease and the site of the anastomosis was broadened by simply apposing the proximal and distal small bowel loops adjacent to the anastomosis with interrupted extra-mucosal absorbable sutures. The constrictive distal jejunal band was divided and removed. The patient made an uneventful post-operative recovery with a return of normal bowel function within three days. On follow-up three months post-operatively, she was well and asymptomatic.

## Discussion

We report a rare case of a small bowel obstruction secondary to a volvulus of a side-to-side jejunoileal anastomosis for bariatric surgery. Between 1960 and 1980, when jejunoileal bypass surgery was common, the mainstay of delayed complications was related to malabsorption of vitamins, hepatic disease and nephrolithiasis [3]. Small bowel obstruction secondary to jejunoileal bypass surgery has been reported but this is usually functional or secondary to adhesions [4,5]. Our case is rare because the volvulus was secondary to the fashioning of a narrow side-to-side anastomosis at initial surgery, whereas all other reported cases have been as a result of a volvulus of an end-to-end or side-to-end anastomosis [5,6]. As in our case, the clinical presentation of a small bowel obstruction following jejunoileal bypass surgery is usually non-specific and is delayed usually by 18 to 60 months [5,6]. As seen in our case, the delay in presentation after the initial surgery can result in diagnostic difficulty. The inherent difficulty in the clinical cause of small bowel obstruction places a great onus on imaging to determine a diagnosis. The literature reports 71% to 92% specificity and 71% to 94% sensitivity of CT scanning in the diagnosis of small bowel obstruction [7,8]. In closed loop obstruction, the specificity falls to 78% [9]. In our case there was a delay in diagnosis due to intermittency of symptoms. A CT scan provided a diagnosis which was subsequently definitively made with an oral contrast swallow study. The importance of early imaging in such cases is crucial in instituting appropriate management. The placement of a proximal jejunal band is uncommon in typical jejunoileal bypass procedures. There are isolated case reports of band-related bowel obstruction; however these are a result of intraluminal band obstruction following erosion of the gastric band [10,11]. Our case is interesting as our findings showed that the jejunal band had migrated into the small bowel mesentery. We describe an effective operating technique for the management of a volvulus secondary to a narrow side to side jejunoileal anastomosis. The fixation of the apex of the volvulus, by approximation of the adjacent bowel proximal and distal to the anastomosis avoided the need to re-fashion the anastomosis. The laparoscopic surgical options in our case were extremely limited by the emergency presentation as well as the previous open surgery. Adhesions post open-abdominal surgeries are well reported [12,13]. They are associated with increased complications and re-hospitalization rates, making laparoscopic intervention difficult in an emergency setting [14]. If such contraindications are not present our technique is quite amenable to a laparoscopic approach. Our case raises several important dilemmas with regard to health tourism. Jejunoileal bypass surgery for bariatric patients has fallen out of favor in European and North American surgical centers due to poor long term outcomes and delayed complications [3,15]. As a result the complications are not commonly encountered and make diagnosis and management difficult. In addition, the surgical center was on another continent making communication and notes requesting challenging. We suggest that perhaps patients who undergo similar complex operations should be advised or provided with a detailed summary of their initial procedure if travelling abroad.

## Conclusions

We describe a case with an important complication of bariatric jejunoileal bypass surgery, now uncommon in western centers. The management of our patient highlights the importance of early appropriate imaging in cases with subacute small bowel obstruction following complex obesity surgery. We describe a simple and effective surgical technique that secures and widens the anastomosis without having to contaminate the peritoneum by enterotomy.

## Consent

Written informed consent was obtained from the patient for publication of this case report and any accompanying images. A copy of the written consent is available for review by the Editor-in-Chief of this journal.

## Competing interests

The authors declare that they have no competing interests.

## Authors' contributions

PHP was the major contributor in writing and editing the manuscript, AS edited the manuscript, PHP, AAPS and KTN performed the primary surgical procedure. All authors read and approved the final manuscript.

## References

[B1] Griffen WO Jr Bivins BA Bell RM The decline and fall of the jejunoileal bypass Surg Gynecol Obstet 1983 157 301 308 6623319

[B2] Bennett JMH Mehta S Rhodes M Surgery for morbid obesity Postgrad Med J 2007 83 8 15 17267672 10.1136/pgmj.2006.048868 PMC2599972

[B3] Requarth JA Burchard KW Colacchio TA Stukel TA Mott LA Greenberg ER Weismann RE Long-term morbidity following jejunoileal bypass. The continuing potential need for surgical reversal Arch Surg 1995 130 318 325 10.1001/archsurg.1995.01430030088018 7887801

[B4] Scott-Conner CE Coil JA Jr Obstruction of defunctionalized loop ten years after jejunoileal bypass for morbid obesity South Med J 1988 81 96 97 10.1097/00007611-198801000-00023 3336808

[B5] Weiner P Shlum H Ganam R Plavnick L Colonic pseudo-obstruction: a late complication of jejunoileal bypass Isr J Med Sci 1984 20 405 406 6547930

[B6] Barry RE Benfield JR Nicell P Bray GA Colonic pseudo-obstruction: a new complication of jejunoileal bypass Gut 1975 16 903 908 10.1136/gut.16.11.903 1238312 PMC1413130

[B7] Scaglione M Grassi R Pinto A Giovine S Gagliardi N Stavolo C Romano L Positive predictive value and negative predictive value of spiral CT in the diagnosis of closed loop obstruction complicated by intestinal ischemia Radiol Med (Torino) 2004 107 69 77 15031698

[B8] Beall DP Fortman BJ Lawler BC Regan F Imaging bowel obstruction: a comparison between fast magnetic resonance imaging and helical computed tomography Clin Radiol 2002 57 719 724 10.1053/crad.2001.0735 12169282

[B9] Mallo RD Salem L Lalani T Flum DR Computed tomography diagnosis of ischemia and complete obstruction in small bowel obstruction: a systematic review J Gastrointest Surg 2005 9 690 694 10.1016/j.gassur.2004.10.006 15862265

[B10] Lantsberg L Kirshtein B Leytzin A Makarov V Jejunal obstruction caused by migrated gastric band Obes Surg 2008 18 225 227 10.1007/s11695-007-9380-z 18163193

[B11] Taskin M Zengin K Unal E Intraluminal duodenal obstruction by a gastric band following erosion Obes Surg 2001 11 90 92 10.1381/096089201321454178 11361175

[B12] Thompson J Pathogenesis and prevention of adhesion formation Dig Surg 1998 15 153 157 10.1159/000018610 9845579

[B13] Schafer ML Krahenb HL Buchler MW Comparison of adhesion formation in open and laparoscopic surgery Dig Surg 1998 15 148 152 10.1159/000018609 9845578

[B14] Parker MC Wilson MS Menzies D Sunderland G Clark DN Knight AD Crowe AM Surgical and Clinical Adhesions Research (SCAR) Group The SCAR-3 study: 5-year adhesion-related readmission risk following lower abdominal surgical procedures Colorectal Dis 2005 7 551 558 10.1111/j.1463-1318.2005.00857.x 16232234

[B15] McFarland RJ Gazet JC Pilkington TR A 13-year review of jejunoileal bypass Br J Surg 1985 71 81 87 10.1002/bjs.1800720202 3971127

